# Oxygen enrichment protects against intestinal damage and gut microbiota disturbance in rats exposed to acute high-altitude hypoxia

**DOI:** 10.3389/fmicb.2023.1268701

**Published:** 2023-10-12

**Authors:** Qianqian Ma, Jiaojiao Ma, Jinxiu Cui, Chenxu Zhang, Yuanzhe Li, Juan Liu, Kangning Xie, Erping Luo, Chi Tang, Mingming Zhai

**Affiliations:** ^1^The College of Life Sciences, Northwest University, Xi’an, Shaanxi, China; ^2^School of Biomedical Engineering, Fourth Military Medical University, Xi’an, Shaanxi, China; ^3^Shaanxi Provincial Key Laboratory of Bioelectromagnetic Detection and Intelligent Perception, Xi’an, Shaanxi, China

**Keywords:** gut microbiota, oxygen enrichment, 16srRNA, intestinal injury, acute high-altitude

## Abstract

Acute high-altitude hypoxia can lead to intestinal damage and changes in gut microbiota. Sustained and reliable oxygen enrichment can resist hypoxic damage at high altitude to a certain extent. However, it remains unclear whether oxygen enrichment can protect against gut damage and changes in intestinal flora caused by acute altitude hypoxia. For this study, eighteen male Sprague–Dawley rats were divided into three groups, control (NN), hypobaric hypoxic (HH), and oxygen-enriched (HO). The NN group was raised under normobaric normoxia, whereas the HH group was placed in a hypobaric hypoxic chamber simulating 7,000 m for 3 days. The HO group was exposed to oxygen-enriched air in the same hypobaric hypoxic chamber as the HH group for 12 h daily. Our findings indicate that an acute HH environment caused a fracture of the crypt structure, loss of epithelial cells, and reduction in goblet cells. Additionally, the structure and diversity of bacteria decreased in richness and evenness. The species composition at Phylum and Genus level was characterized by a higher ratio of *Firmicutes* and *Bacteroides* and an increased abundance of *Lactobacillus* with the abundance of *Prevotellaceae_NK3B31_group* decreased in the HH group. Interestingly, after oxygen enrichment intervention, the intestinal injury was significantly restrained. This was confirmed by an increase in the crypt depth, intact epithelial cell morphology, increased relative density of goblet cells, and higher evenness and richness of the gut microbiota, *Bacteroidetes* and *Prevotellaceae* as the main microbiota in the HO group. Finally, functional analysis showed significant differences between the different groups with respect to different metabolic pathways, including Amino acid metabolism, energy metabolism, and metabolism. In conclusion, this study verifies, for the first time, the positive effects of oxygen enrichment on gut structure and microbiota in animals experiencing acute hypobaric hypoxia.

## 1. Introduction

Acute exposure is defined as the sudden transition from lowland areas to plateau regions situated at altitudes exceeding 2,500 m above sea level. During acute hypoxic altitude exposure, the body is temporarily unable to adapt to the environment, leading to physiological discomfort. More than 50% of people experience acute reactions, such as nausea, vomiting, diarrhea, and abdominal distension during plateau exposure (Meier et al., 2017). These symptoms appear quickly and show a dose-response relationship, meaning that the more severe the hypoxia, the stronger the response. Additionally, a qualitative change over time may result in acute injury if the body’s compensatory mechanisms are unable to respond in time (Nuss, 2022). Hypoxia is the primary driving force of injury during high-altitude exposure. Reduced inspiratory oxygen levels at high altitudes can affect normal physiological processes throughout the body, leading to irreversible damage. When the body enters a hypoxic environment, the nervous, cardiovascular, respiratory, and digestive systems are damaged to varying degrees (Adak et al., 2014; Abe et al., 2017; Bonkowsky and Son, 2018; Pan et al., 2022).

The external environment affects the gut environment. In particular, the gut microbiota is highly sensitive to hypoxia. Plateau hypoxia leads to severe gastrointestinal dysfunction (Luks et al., 2017). Together, biological, chemical, mechanical, and immune barriers constitute a complete intestinal defense system (Verdu et al., 2015). Among them, the biological barrier dominated by gut microbiota is considered a key “organ” that substantially contributes to the health of the host body and plays an important biological function (Li et al., 2016). In general, the host and gut microbes maintain a dynamic coexistence and a mutually beneficial relationship that may be modified under external pressure. These changes are commonly accompanied by the emergence and development of disease. In recent years, intensive studies of the gut microbiome after exposure to hypoxia have revealed a strong correlation between the composition and diversity of the gut microbiome and the pathological state of the body. Studies have shown that after exposure to high-altitude hypoxia at 5,000 m, rats develop pathological cardiac hypertrophy accompanied by significant changes in the structure and diversity of the gut microbiota (Pan et al., 2022). In a study on the intestinal microbiota of Tibetan people with hypertension and healthy individuals in high-altitude areas, it was found that the gut microbiota of patients with hypertension exhibited high diversity, and the microbiota colonies differed significantly from those of healthy individuals. Additionally, the species composition was significantly different from that of healthy individuals, as demonstrated by the elevation of Verrucomicrobia and Akkermansia (Zhu et al., 2020). A consistent finding of these studies is the strong correlation between the gut microbiome and host health in plateau hypoxia. Therefore, there is a critical need to find effective ways to maintain the homeostasis of gut microbes in hypoxic environments at high altitudes. However, currently, there is no effective method for preventing and treating gut microbiome disturbances in hypoxia-depleted environments.

Oxygen enrichment intervention is a common and effective measure to improve altitude sickness. Existing studies have shown that the equivalent physiological altitude decreases by 300 m for every 1% increase in oxygen concentration (West, 2015). Enriching the air with oxygen can improve cognitive and motor functions, such as reaction time and eye-hand coordination, and enhance the individual’s sense of efficacy and wellbeing (Gerard et al., 2000). In addition, oxygen-enriched physiotherapy may have positive psychological effects on diseases related to plateau hypoxia (Hasler et al., 2020). However, it is unclear whether oxygen enrichment can improve and protect against intestinal damage and microbiota imbalance during acute HH exposure. The objective of this study was to investigate the potential protective effects of oxygen enrichment on intestinal damage and microbiota under acute hypobaric hypoxic conditions.

In this study, a rat model was established in a hypobaric hypoxia chamber simulating an altitude of 7,000 m, and an oxygen-enriched intervention was performed. The effects of oxygen enrichment on the gut tissue and microbiota of the rats were investigated using histomorphological and bioinformatics analyses. The present study aimed to characterize the potential effects of oxygen enrichment on protecting the gut tissue and regulating the intestinal microbiota following acute hypobaric hypoxic exposure.

## 2. Materials and methods

### 2.1. Animals and experimental design

**FIGURE 1:**
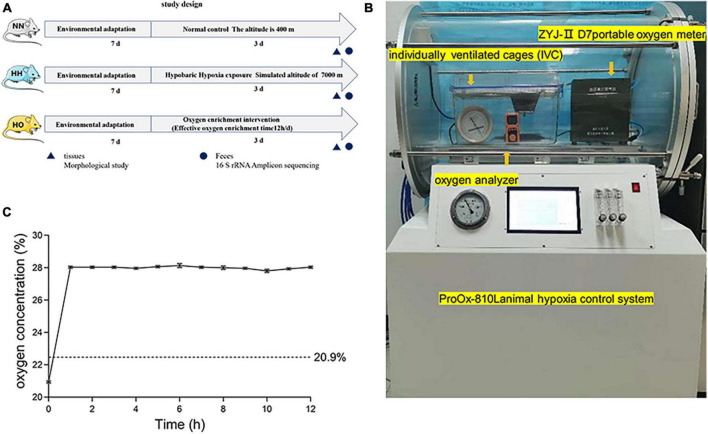
The experimental equipment and scheme used in this study. **(A)** The flow chart of the experimental scheme used in this study. **(B)** In a hypobaric anoxic chamber, a local oxygen-enriched environment is constructed by combining a portable oxygen enrichment device with a separately ventilated cage (IVCs). **(C)** The oxygen concentration of the oxygen enrichment device in the gas produced at the simulated height (7,000 m).

The experimental design is shown in Figure 1A. Seven-week-old male Sprague–Dawley rats were obtained from the Animal Center of the Fourth Military Medical University. The experimental procedures were approved by the Institutional Animal Care and Use Committee of the Fourth Military Medical University and were strictly performed according to the guidelines of the Care and Use of Laboratory Animals published by the National Institutes of Health (Kilkenny et al., 2010). The animals were kept at a controlled temperature of 23°C ± 1°C with a light-dark cycle of 12 h. Food and water was freely available to the rats throughout the experiment.

Eighteen rats were randomly divided into the normal control (NN, *n* = 6), hypobaric hypoxic (HH, *n* = 6), and hypobaric oxygen-enriched (HO, *n* = 6) groups. The NN group was placed at normoxic conditions at an altitude of 400 m (Xi’an, China), the group HH was placed at an HH chamber (ProOx-810L, Shanghai Tawang Intelligent Technology Co., Ltd., China), equivalent altitude of 7,000 for 72 h. The HO group used oxygen-enriched equipment (Figure 1B) for oxygen-enriched intervention (12 h per day). The device consists of a portable oxygen-enriched instrument and an independent ventilation cage (IVC) for rats. The temperature, feeding condition and feeding type during molding were consistent with the control group. To minimize experimental errors caused by opening the cabin, we provided ample food and water that the animals could access freely until the end of the modeling. This setup allowed the creation of a local oxygen-enriched environment within the plateau hypoxia simulation chamber. The detailed principle of the device was previously described in the research conducted by Shen et al. (2013). After completing the assembly, an oxygen analyzer (OXYMAT61, Siemens, Erlangen, Germany) was used to measure the oxygen concentration in the IVC. Changes in the oxygen concentration in the local oxygen-enriched environment were then detected at a simulated altitude of 7,000 m. From the beginning of the portable oxygen machine, the oxygen concentration in the IVC was recorded every hour for 12 h (Supplementary Table 1). After three separate trials, the results were plotted as line graphs (Figure 1C). The oxygen-enriched environment test showed that the oxygen concentration in the medium remains stable at 28 ± 0.2% every 12 h.

### 2.2. Sample collection

Rats were euthanized at designated time points using a sodium pentobarbital solution (1%, 50 mg/kg), and colon tissue and stool samples were collected immediately. Subsequently, these colon tissue and stool samples were transferred to a −80°C refrigerator for short-term storage prior to testing.

### 2.3. Histological analysis

The colon was cut into 1 mm thick slices, fixed with 4% tricresol at 4°C for 24 h, dehydrated with 95% ethanol, and embedded with paraffin. The sample was then cut into 4 micron slices and the slices were stained with hematoxylin-eosin (H&E). The prepared sections were stained with PAS. Gradient dewaxing of sections with xylene and rehydrated ethanol. After treatment with 1% periodate acid (Servicebio) for 15 min, wash in tap water, wash twice in distilled water, and soak in Schiff’s reagent (Servicebio) for 30 min under photoprotection. Images are obtained using a computer supported imaging system connected to an optical microscope (VS200, Olympus, Japan). Five non-repetitive visual fields were randomly selected for each sample, goblet cell count and crypt depth analysis were performed using Image J.

### 2.4. DNA extraction and sequencing

The composition of fecal microbiota was analyzed in the NN, HH and HO groups. Fecal samples (*n* = 6) were collected and immediately placed in a sterile cryopreserved tube at −80°C, and subsequent analysis was carried out immediately after the whole experiment. Microbial DNA was extracted using a HiPure Stool DNA Kit (Magen, Guangzhou, China) following the manufacturer’s protocol. The total DNA was quantified using a NanoDrop 2000 spectrophotometer (Thermo Fisher Scientific, Waltham, MA, USA). The PCR was carried out in a 50 μL reaction volume using TransGen High-Fidelity PCR SuperMix (TransGen Biotech, Beijing, China), 0.2 μM forward and reverse primers, and 5 ng template DNA. The full-length 16S rRNA was amplified by PCR (95°C for 2 min, followed by 35 cycles of 95°C for 30 s, 60°C for 45 s, and 72°C for 90 s, with a final extension at 72°C for 10 min). The primer set targeted the V3 and V4 hypervariable regions of the 16S rRNA gene using 341F (5-CCTACGGGNGGCWGCAG-3) and 806R (5-GGACTACHVGGGTATCTAAT-3) primers.

Using FASTP (version 0.18.0) (Chen et al., 2018) to obtain high-quality clean reads, the paired-end clean reads were then merged into the original tags using FLASH (version 1.2.11) (Magoè and Salzberg, 2011). The noise sequences of the original tags were then filtered under specific conditions (Bokulich et al., 2013) to obtain high-quality clean tags. Subsequently, the clean tags were clustered into operational taxonomic units (OTUs) with a similarity of ≥ 97% using the UPARSE pipeline (version 9.2.64) (Edgar, 2013). Finally, the UCHIME algorithm (Edgar et al., 2011) removed all chimeric labels and obtained valid labels for further analysis. The marker sequence with the highest abundance was selected as the representative for each cluster. Sequencing was performed using the Illumina MiSeq system (Illumina, San Diego, CA, USA), following the manufacturer’s instructions.

### 2.5. Bioinformatics analysis

Representative OTU sequences were classified into organisms using a naïve Bayesian model, the RDP classifier (version 2.2) (Wang et al., 2007), based on the SILVA database (version 138.1) (Pruesse et al., 2007). The confidence threshold value was set to 0.8. The abundance statistics for each taxon were visualized using Krona (version 2.6) (Ondov et al., 2011). Community composition was visualized using a stacked bar plot in the ggplot2 package (version 2.2.1) of R (Robert et al., 2018). Shannon, Simpson, and Good’s Coverage indices were calculated using QIIME (version 1.9.1). Principal component analysis (PCoA) was performed using Vegan (version 2.5.3) in R software. Kyoto Encyclopedia of Genes and Genomes pathway analysis of OTUs was inferred using PICRUSt (version 2.1.4) (Langille et al., 2013).

### 2.6. Statistical analysis

Data are expressed as the mean ± standard error of the mean. The figures were prepared using GraphPad Prism 9.4 software; comparisons between groups were performed using GraphPad Prism 9.4 software using a one-way analysis of variance. The Tukey-HSD test was used to compare the differences α-diversity between the three groups. PCoA based on the abundances of each sample was performed to evaluate the degree of similarity between the samples. LEfse software was used to analyze the difference groups. First, kruskal-Wallis test was performed among samples from all groups, and then wilcoxon test was used to compare the selected different species between the two groups. Linear Discriminant Analysis (LDA) was used to select differences whose score ≥ 3. Differences were considered statistically significant at a *p*-value < 0.05.

## 3. Results

### 3.1. Changes in intestinal morphology after acute high-altitude hypoxia exposure and oxygen-enriched intervention

**FIGURE 2:**
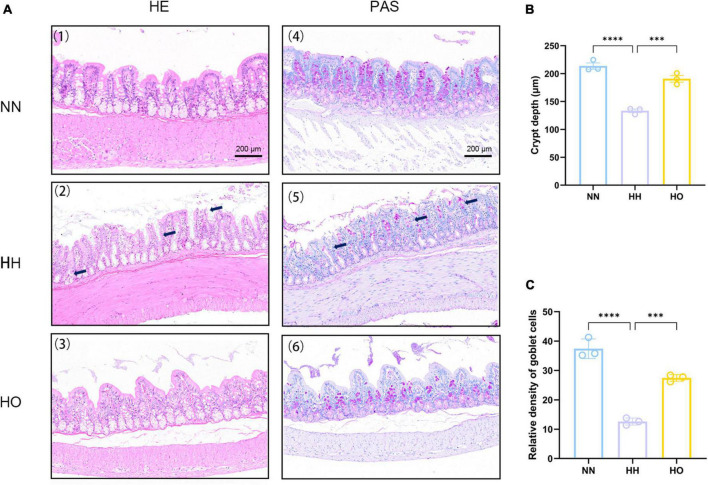
Effects of acute high-altitude hypoxia and oxygen enrichment on colonic histology in rats. **(A)** HE staining and PAS staining were used to detect the effects of acute high altitude hypoxia and hyperoxia on colon crypt structure and goblet cells in rats. (1) The colonic HE sections of the control group showed normal crypt structure, (2) the colonic HE sections of rats in the 7,000 m acute altitude hypoxia group were stained, and (3) the colonic HE sections of rats in the oxygen enriched group were stained. (4) colonic PAS staining results of the control group, (5) colonic PAS staining results of 7,000 m acute altitude hypoxia group, and (6) colonic PAS staining results of oxygen enriched group. **(B)** The depth of colon recess in rats, and **(C)** the relative density of colonic cups in rats. Data were expressed as the mean ± SEM (*n* = 3). ****p < 0.0001, ***p < 0.001.

The intestinal morphological damage was assessed during the period of plateau hypoxia exposure, and the results are presented in Figure 2. Hematoxylin and eosin staining (Figure 2A) revealed that the colonic tissue of rats in the NN group exhibited a normal appearance with intact epithelial cells, straight intestinal crypts, and closely arranged intestinal glands, indicating a clear structure. In contrast, the colonic tissue of rats exposed to acute high-altitude hypoxia at 7,000 m showed deformation and reduced depth of crypts (Figure 2B and Supplementary Table 2). This suggests that acute high-altitude exposure resulted in changes in colonic cell morphology and reduced crypt depth, indicating compromised intestinal epithelium.

The relative density of goblet cells (Supplementary Table 3) was determined after acute plateau hypoxia exposure and oxygen enrichment (Figure 2C). We observed a significant decrease in the relative density of intestinal goblet cells following acute hypoxic exposure compared to the NN group. This reduction in goblet cells indicates a disruption of the mucus layer, worsening intestinal damage. Conversely, after oxygen enrichment, the relative density of goblet cells significantly increased, as shown in Figure 2C. Surprisingly, the oxygen-enriched intervention resulted in neatly arranged and tightly packed colon cells, partial recovery of epithelial cell and crypt morphology, and increased relative density of goblet cells. These results suggest that the oxygen-enriched intervention alleviated intestinal morphology damage to a certain extent under hypoxic conditions at altitude compared to the HH group.

### 3.2. Sequencing analysis, richness, and diversity of rat gut microbiota

After quality control filtering, a total of 1,971,986 Raw tags were obtained, with an average of 109,555 ± 20,883 sequences per sample, and 1,962,388 Clean tags were obtained, with an average of 109,022 ± 20,779 sequences per sample. After the removal of chimeras, a total of 1,558,956 sequences of Effective tags were obtained, with an average of 86,609 ± 15,990 sequences per sample, 18,960 OTUs were coclustered, with an average of 1,053 ± 155 OTUs per sample (Supplementary Table 4).

**FIGURE 3:**
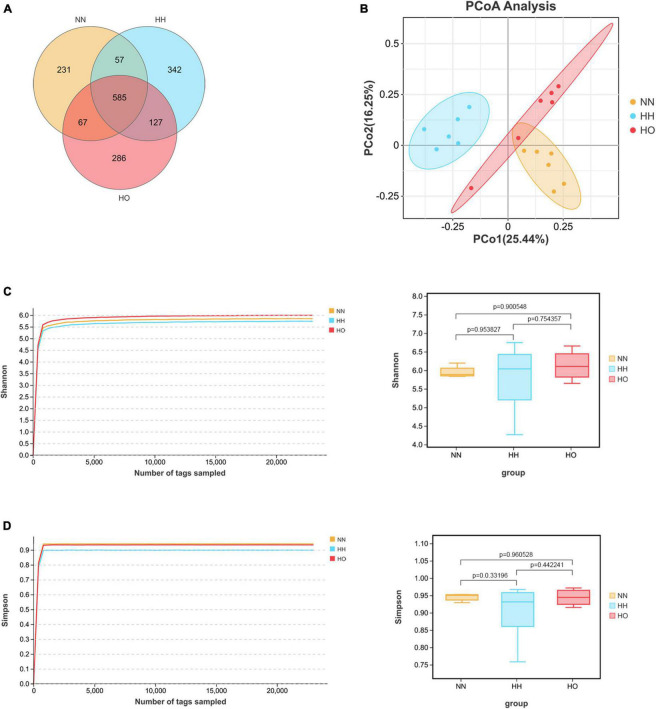
Sequencing analysis, richness and diversity of gut microbiota in rats. Wayne diagram analysis is carried out according to OTU abundance information to understand the common or unique information of OTU between different samples or groups **(A)**; PCoA principal coordinate analysis **(B)** at OTU level. Shannon dilution curve and index **(C)**, Simpson dilution curve and index **(D)**. The box plot of α diversity index was drawn by Tukey-HSD test for two or two groups of post-test after comparing multiple groups.

The coverage value of the gut microbiota in the three groups of rats (Supplementary Figure 1) approached 1 when the sequencing volume was 1,000. As the sample sequencing reads increased, the coverage value remained unchanged, indicating that each sample was adequately detected, and that the sequencing depth sufficiently covered the species. This suggests that fewer undetected species needed to be identified, thus confirming the reliability of our data. A Venn diagram (Figure 3A) illustrates that 1,695 OTUs were detected at the OTU level. Among these, the HH group had the highest number of unique OTUs (342), followed by the HO group (286), and the NN group had the lowest number (231). The Shannon dilution curve (Figure 3C), Simpson dilution curve (Figure 3D) gradually flattened when the sequencing reads was 1,000 and did not fluctuate with an increase in the sample sequencing reads. The Tukey-HSD test method was used to perform two or two sets of post-test. Shannon index (Figure 3C) and Simpson index (Figure 3D) was not obvious change. This observation reflects the diversity of gut microbiota among the three groups of rats. The difference in curve height indicates the alpha diversity of the communities across different samples. Acute high-altitude hypoxia exposure affected the structure of colonic microbial communities in rats. The species richness of the NN and HO groups was higher than that of the HH group. PCoA at the OTU level (Figure 3B) revealed clear clustering patterns among samples from the NN, HH, and HO groups. This suggests that there were significant differences in the gut microbiota among the three groups of rats, with minimal variation within each group. The similarity between the gut microbiota samples of rats in different groups was low, whereas the similarity between the gut microbiota samples of rats within the same group was high.

### 3.3. Differences in gut microbiota composition in rats after acute high-altitude hypoxia exposure and oxygen-enriched

**FIGURE 4:**
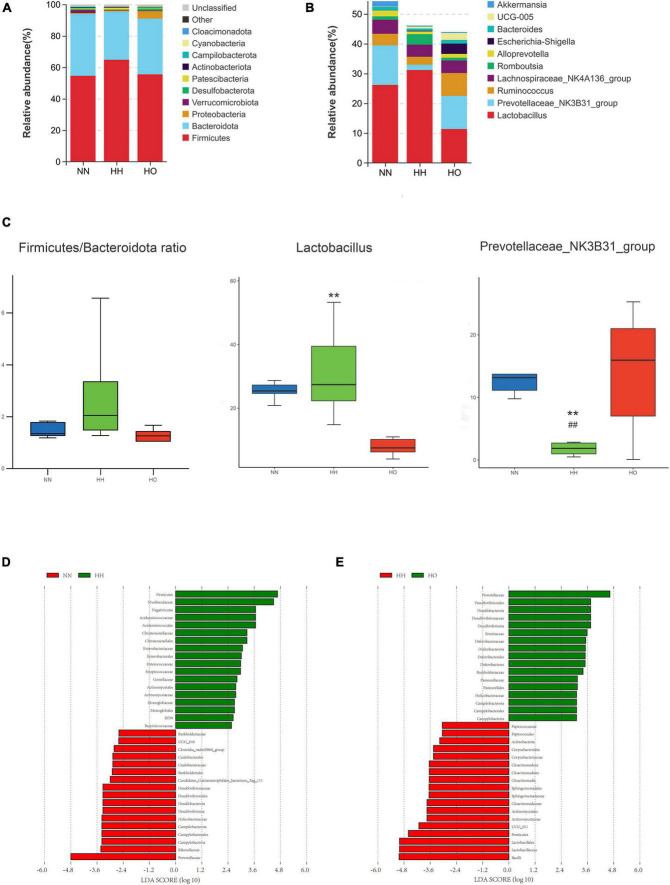
Analysis of gut microbiota structure and differential bacteria in rats. **(A)** Phylum level; **(B)** genus level. **(C)** The Firmicutes/Bacteroidetes ratio, the relative abundance diagrams of genus level Lactobacillus and Prevotellaceae_NK3B31_group, the differences between groups were analyzed using Maaslin2. Differences in microbial taxas was calculated by LEfSe. linear discriminant analysis score ≥ 3. **(D)** NN vs. HH, **(E)** HH vs. HO. All data are expressed as mean ± SEM (*n* = 6). # be used to represent NN vs. HH, * be used to represent HH vs. HO. ^##^*p* < 0.01, **p < 0.01.

Based on the species annotation results, the top ten species within each group were selected at the phylum level of the gut microbiota. These species were then represented using a column chart. Figure 4A shows the differences in the percentage composition of the gut microbiota in each group. The top ten bacterial phyla in terms of species abundance at the phylum classification level are *Firmicutes, Bacteroidetes, Proteobacteria, Verrucomicrobia, Desulfobacteria, Patescibacteriateria, Actinobacteria, Campybacteria Campylobacterota, Cyanobacteria*, and *Cloacimonadota*. Among them, the ratio of *Firmicutes/Bacteroidetes* in the HH group increased (Figure 4B). Although it was not statistically significant, it may be related to the imbalance of gut microbiota.

Based on the results of the species annotation, the species with the top 10 relative abundances at the genus level of the gut microbiota were selected, and the differences in the percentage composition of intestinal bacteria in each group are shown in the column chart in Figure 4C. The top ten species at the genus level were *Lactobacillus, Prevotellaceae_NK3B31_group, Ruminococcus, Lachnospiraceae_NK4A-136_group, Romboutsia, Alloprevotella, Escherichia-Shigella, Bacteroides, UCG-005* and *Akkermansia*. Among these, bacteria with a species abundance of more than 2% were *Lactobacillus, Prevotella_NK3B31_group, Ruminococcus*, and *Lachnospiraceae_NK4A-136_group*. The abundance of *Lactobacillus* was significantly lower in the HO group than in the HH group. Similarly, the species abundance of the *Prevotella_NK3B31_group* significantly decreased in the HH group compared to the NN group, but increased in the HO group compared to the HH group. Additionally, the abundance of *Ruminococcus* decreased in the HH group compared to that in the NN group and increased in the HO group, although this difference was not statistically significant.

To further understand the effect of oxygen enrichment on gut microbiota after acute hypoxia exposure at high altitudes, we analyzed the enriched microbiota by LEfSe (Linear discriminate analysis size effect) in order to compare the three groups, so as to find the species with significant differences in abundance between groups, which made the results more biologically significant. At the phylum-to-family level, *Prevotellaceae*, *Rikenellaceae* and *Campylobacteria* were the main taxa in the NN group; *Firmicutes*, *Muribaculaceae*, and *Negativicutes* were the focal taxa in the HH group (Figure 4D); and *Desulfobacterota* and *Prevotellaceae* were the core taxa in the HO group (Figure 4E).

### 3.4. Prediction of gut microbiota function in rats after acute high-altitude hypoxia exposure and oxygen enrichment intervention

**FIGURE 5:**
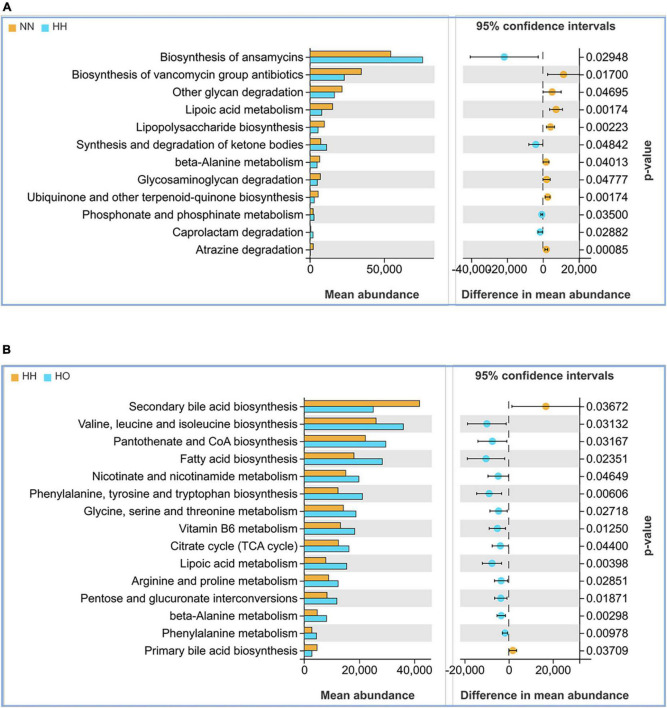
Functional analysis of the gut microbiota in each group of rats. **(A)** NN vs. HH, **(B)** HH vs. HO.

Changes in the structure and composition of the gut microbiota are often accompanied by alterations in related biological processes. Accordingly, we utilized the PICRUSt2 software to predict the gene functions of the enriched bacterial communities, and the results revealed distinct differences in the gene function predictions of the gut microbiota in the three groups of rats. Compared to the NN group, the HH group exhibited increased expression abundance of antibiotic synthesis (*p* = 0.02948) and ketone body synthesis and degradation (*p* = 0.04842). Conversely, the HH group showed decreased expression in lipoic acid metabolism (*p* = 0.00174), β-alanine metabolism (*p* = 0.04013), glycosaminoglycan degradation (*p* = 0.04777), and phosphonate and hypophosphonate metabolism (*p* = 0.00174) (Figure 5A). Compared to the HH group, the HO group showed decreased expression of genes associated with primary bile acid metabolism (*p* = 0.03709) and the secondary bile acid pathway (*p* = 0.03276). Additionally, there was an increase in the expression abundance of gene functions involved in the biosynthesis of valine, leucine, and isoleucine (*p* = 0.03132). Furthermore, certain cellular metabolic pathways, such as the biosynthesis of pantothenic acid and coenzyme A (*p* = 0.03197), tricarboxylic acid cycle (*p* = 0.04400), and lipoic acid metabolism (*p* = 0.00398), were significantly enhanced (Figure 5B). These findings suggest that changes in the metabolic pathways mediated directly or indirectly by the gut microbiota may be closely related to the health status of the host.

## 4. Discussion

High altitudes have been shown to influence the composition and diversity of both human and animal gut microbiota (Lan et al., 2017; Zhao et al., 2018; Jia et al., 2020; Bai et al., 2022). However, during field investigations, it is challenging to isolate the impact of geographical environment, diet, species differences, individual variations, and genetic factors on the gut microbiota (Li and Zhao, 2015; Matey-Hernandez et al., 2018; Netto Candido et al., 2018). To address this, researchers have conducted controlled laboratory experiments and found that hypobaric hypoxia significantly affects gut microbiota (Maity et al., 2013; Adak et al., 2014). Despite this, there is limited information on the specific characteristics of intestinal microorganisms during acute hypobaric hypoxia challenges, and less attention has been paid to regulating the gut microbiota to mitigate the risk of acute injury. This study reveals that hypobaric hypoxia disrupts the morphological structure of the gut and alters the gut flora. Following oxygen enrichment intervention, the gut flora exhibited higher uniformity and richness compared to the HH group, indicating successful regulation of the gut flora balance.

The structural integrity of the intestine is fundamental to intestinal health. acute exposure to hypoxia resulted in severe structural damage to the colon tissue. The results demonstrated the reduced depth of the crypt structure, shedding of epithelial cells, and a decrease in goblet cells. Crypt depth is a key indicator of intestinal health (Liu et al., 2019). intestinal glands are responsible for forming new intestinal cells, including epithelial, goblet, and mast cells, which play important roles in maintaining epithelial stability, stabilizing the intestinal environment, and regulating defense responses (Barker et al., 2007; Shi et al., 2017). Our study observed a reduction in the number of goblet cells following acute exposure to high-altitude hypoxia. Previous research has emphasized the crucial role of goblet cells in regulating the intestinal barrier in both healthy and diseased states (Birchenough et al., 2016; Bergstrom et al., 2020; Grondin et al., 2020). The reduction in goblet cells led to a decrease in intestinal mucus secretion, resulting in thinning of the intestinal mucus layer. This thinning increases the permeability of the intestinal barrier, creating more opportunities for colonization by endotoxins and pathogens, and subsequently increasing the risk of infection and disease (Gustafsson and Johansson, 2022). As hypothesized, oxygen enrichment resulted in a notable increase in the relative density of goblet cells. This increase contributes to the fortification of the intestinal epithelium and mucus layer, establishing a physical barrier against potential pathogens. Goblet cells are essential in protecting against toxic substances in the intestinal cavity and lower tissues. Epithelial damage, particularly when it affects the protective properties of the secretory products of goblet cells, has the potential to induce inflammation (Van der Sluis et al., 2006). Morphological studies confirmed the damage to the intestinal mucosa caused by acute hypobaric hypoxia. However, the introduction of oxygen counteracted the injury by preserving the structure of the intestinal crypt and increasing the number of goblet cells compared to the HH group. Oxygen enrichment has demonstrated its ability to alleviate this type of injury.

To explore the potential association between oxygen-enriched protection and the gut microbiome, we employed high-throughput 16s rRNA gene sequencing to investigate the complexity of gut microbial communities, including community diversity, composition, and richness. The results of our study demonstrated that the intestinal microbiota of rats exhibited higher richness and evenness after oxygen enrichment intervention compared to the HH group. This increase in microbial diversity may be associated with the ability to resist imbalances in the intestinal microbiota under acute hypobaric hypoxia. Walker et al. (2011) highlighted that a decrease in intestinal microbiota diversity is indicative of a gut microbiota imbalance. Additionally, Sommer et al. (2017) suggested that high diversity plays a crucial role in microbial resilience. Therefore, our findings suggest that oxygen enrichment may help restore the diversity of the gut microbiota.

Interestingly, phylum-level species analysis revealed that acute high-altitude hypoxia exposure was accompanied by an increase in the ratio of *Firmicutes/Bacteroidetes*, considered one of the characteristics of ecological disorders. An increase or decrease in the ratio of *Firmicutes/Bacteroidetes* is usually associated with obesity, and the latter is associated with inflammatory bowel disease (Stojanov et al., 2020); however, due to low temperature and environmental pressure, hypoxia exposure at high altitudes is often accompanied by weight loss. This is inconsistent with our results, and we speculate that this may be a way for the body to resist acute hypoxic injury. Most members of the *Bacteroides* family are involved in the fermentation of indigestible oligosaccharides and underutilized carbohydrates in the small intestine to synthesize short-chain fatty acids, which are rich sources of energy for the host (Macfarlane and Macfarlane, 2003; Sartor, 2008). In addition, some studies have reported that the ratio of thick-walled bacteria to *Bacteroides* in Chinese Han people living at high elevations is higher than that in Han people living at low elevations (Lan et al., 2017). Similarly, the gut microbiota of Tibetans changes with increasing altitude, in which *Bacteroides* increased with increasing altitude (Li and Zhao, 2015). The same results were found not only in humans but also in animals; after exposure to high altitudes, *Bacteroides* in the feces of Wistar rats increased significantly (Sun et al., 2020). These results support our conjecture that an increase in *Bacteroides* abundance is one of the characteristics of the gut microbiota after exposure to high altitudes. To resist the imbalance of gut microbiota at high altitudes, oxygen-enriched interventions can regulate the abundance of *Bacteroides*, increase the energy source of the host, and maintain the balance of microbiota to reduce the disturbance of the gut microbiota caused by acute hypobaric hypoxia.

At the genus level, there was an initial increase, followed by a decrease in the species composition of *Lactobacillus*. Specifically, the abundance of *Lactobacillus* significantly increased in the HH group compared with that in the HO group. This trend was contrary to the relative abundances observed for *Prevotellaceae_NK3B31_group* and *Ruminococcus*. Similar results were observed in the analysis of the differential microbiota. A higher relative abundance of *Lactobacillus* was found to contribute to the protection of the intestinal mucosal barrier and enhancement of intestinal function (Xu et al., 2019), indicating that lactic acid bacteria may have a greater ability to adapt to hypoxic environments at high altitudes. To resist adverse effects in a high-altitude hypoxic environment, the gut microbiota of rats may regulate probiotics to maintain species balance. Oxygen enrichment increased the abundance of butyrate-producing bacteria such as *Prevotellaceae_NK3B31_group* and *Ruminococcus. Ruminococcus* is a beneficial parasitic microorganism in the cecum and colon that can degrade various polysaccharides and fibers to produce short-chain fatty acids (Hooda et al., 2012; Donaldson et al., 2016). Butyrate is a short-chain fatty acid that produces ketones and carbon dioxide. It is the main source of energy for colon cells, and a lack of it can lead to damage to the intestinal barrier function (Hamer et al., 2012). *Prevotella* is one of the most abundant species in the gut microbiome (Tett et al., 2021). However, whether its effects on human health are positive or harmful remains controversial. Changes in *Prevotella* abundance as a pathogen are usually associated with periodontitis and rheumatoid arthritis (Scher et al., 2013; Pianta et al., 2017; Sharma et al., 2022). Conversely, several studies have suggested that it is beneficial as a bacterium for glucose homeostasis and host metabolism by improving glucose regulation and metabolic processes (Asnicar et al., 2021). Further research is needed to enhance understanding of the genetic potential of *Prevotella* and its interactions with the host and other bacteria to reveal its regulatory properties for health or disease and potential causal relationships. Although the exact role of *Prevotella* remains to be elucidated, our results suggest that oxygen enrichment can alter the abundance of different microbiota to suit hypobaric hypoxia and regulate the abundance of probiotics to maintain the ecological balance of the gut microbiota.

To further investigate the regulatory effect of oxygen enrichment on the gut microbiota exposed to acute hypobaric hypoxia, we performed a functional prediction of the enriched gut microbiota communities. During acute hypobaric hypoxia, the body undergoes a series of compensatory measures to rapidly adapt to the environment and resist external pressures. These parameters are closely related to physiology and metabolism. The effect of a hypoxic environment on microbial communities at high altitudes is closely linked to energy and cell metabolism. Ketone bodies are primarily used as energy sources in the extrahepatic tissues in the absence of glucose. After ketone bodies are absorbed by extrahepatic tissues, there are two metabolic pathways: one enters the mitochondria for oxidative decomposition, called the oxidative metabolic fate, which is the main metabolic mode, and the other participates in lipid synthesis in the cytoplasm, called the non-oxidative metabolic fate (Newman and Verdin, 2014; Puchalska and Crawford, 2017). Oxygen is the main driving force in high-altitude hypoxic environments, and the use of oxygen is indispensable for energy generation. During acute hypoxia, to meet the normal demand for oxygen, the body uses ketone bodies as the main fuel in the absence of sufficient glycogen supplements, leaving the valuable glucose for red blood cells to maintain the balance of energy metabolism (Veech et al., 2017). The expression of ketone body synthesis and degradation pathway was increased in the HH group, and we obtained the same results as the above study. Westerterp (2001) reported that energy imbalance is associated with reduced food intake and loss of appetite in a high-altitude hypoxic environment, which is a result of metabolic demands exceeding the energy supply and the body trying to cope with acute altitude reactions and other illnesses, resulting in energy imbalance. Notably, the tricarboxylic acid cycle is an essential metabolic pathway. There is growing evidence that this metabolic center plays an important role in counteracting cellular stress by coordinating a wide range of critical biological processes and signaling, and providing metabolites to suppress a wide range of cellular disruptions (MacLean et al., 2023). The expression of tricarboxylic acid cycle pathway genes increased after oxygen enrichment, indicating that oxygen enrichment can meet the energy requirements for metabolism during hypoxia. This intervention helps offset the energy imbalance and reduces hypoxia damage. Amino acids are crucial for nutrition, survival, and development. They also play vital roles in the regulation of material metabolism and information transmission. In a study comparing sterile and conventional mice, researchers found that the latter had a different distribution of free amino acids in the gastrointestinal tract. These findings suggest that the resident species of the gut microbiome are important for maintaining host amino acid homeostasis and overall health (Mardinoglu et al., 2015). Our study showed that oxygen enrichment intervention could potentially rebalance energy and metabolism in individuals exposed to high-altitude hypoxia. This intervention may also help alleviate hypoxic injury by improving energy and amino acid metabolism, which are mediated by the gut microbiota. Notably, various bacteria, including *Bacteroides*, may play significant roles in amino acid metabolism in the large intestine.

In conclusion, this study demonstrated that oxygen enrichment at high altitudes effectively mitigates intestinal injury caused by acute hypoxia. It protects intestinal morphology and maintains the integrity of the intestinal barrier. Furthermore, oxygen enrichment plays a crucial role in regulating the diversity of gut microbiota. This is achieved by modulating the composition and proportion of the gut microbiota and their associated physiological processes, thereby counteracting the acute altitude injury mediated by the gut microbiota. However, this study has certain limitations. First, the current findings do not explain whether oxygen enrichment can counteract hypobaric hypoxic damage by regulating intestinal flora. Further investigations are required to explore the underlying mechanisms, which will be the focus of our future studies. Additionally, more detailed oxygen enrichment intervention parameters may be necessary for effective protection against acute hypoxic injuries at high altitudes.

## Data availability statement

The data presented in the study are deposited in the NCBI repository, BioProject number PRJNA998771 available at: https://www.ncbi.nlm.nih.gov/bioproject/PRJNA998771.

## Ethics statement

Experimental procedures were approved by the Institutional Animal Care and Use Committee of Air Force Medical University and strictly performed according to the guidelines of the Care and Use of Laboratory Animals published by the National Institutes of Health.

## Author contributions

QM: Data curation, Formal analysis, Writing—original draft, Investigation, Methodology, Conceptualization. JM: Investigation, Methodology, Writing—review and editing. JC: Investigation, Software, Writing—review and editing. CZ: Data curation, Investigation, Visualization, Writing—review and editing. YL: Investigation, Methodology, Writing—review and editing. JL: Investigation, Methodology, Writing—review and editing. KX: Software, Writing—review and editing. EL: Investigation, Methodology, Writing—review and editing. CT: Resources, Supervision, Validation, Writing—review and editing. MZ: Resources, Writing—review and editing.
