# Supplementary figures and images for: Oxygen enrichment protects against intestinal damage and gut microbiota disturbance in rats exposed to acute high-altitude hypoxia
Source: Front Microbiol. 2023 Oct 12;14:1268701. doi: 10.3389/fmicb.2023.1268701 (PMC10600524; doi:10.3389/fmicb.2023.1268701)

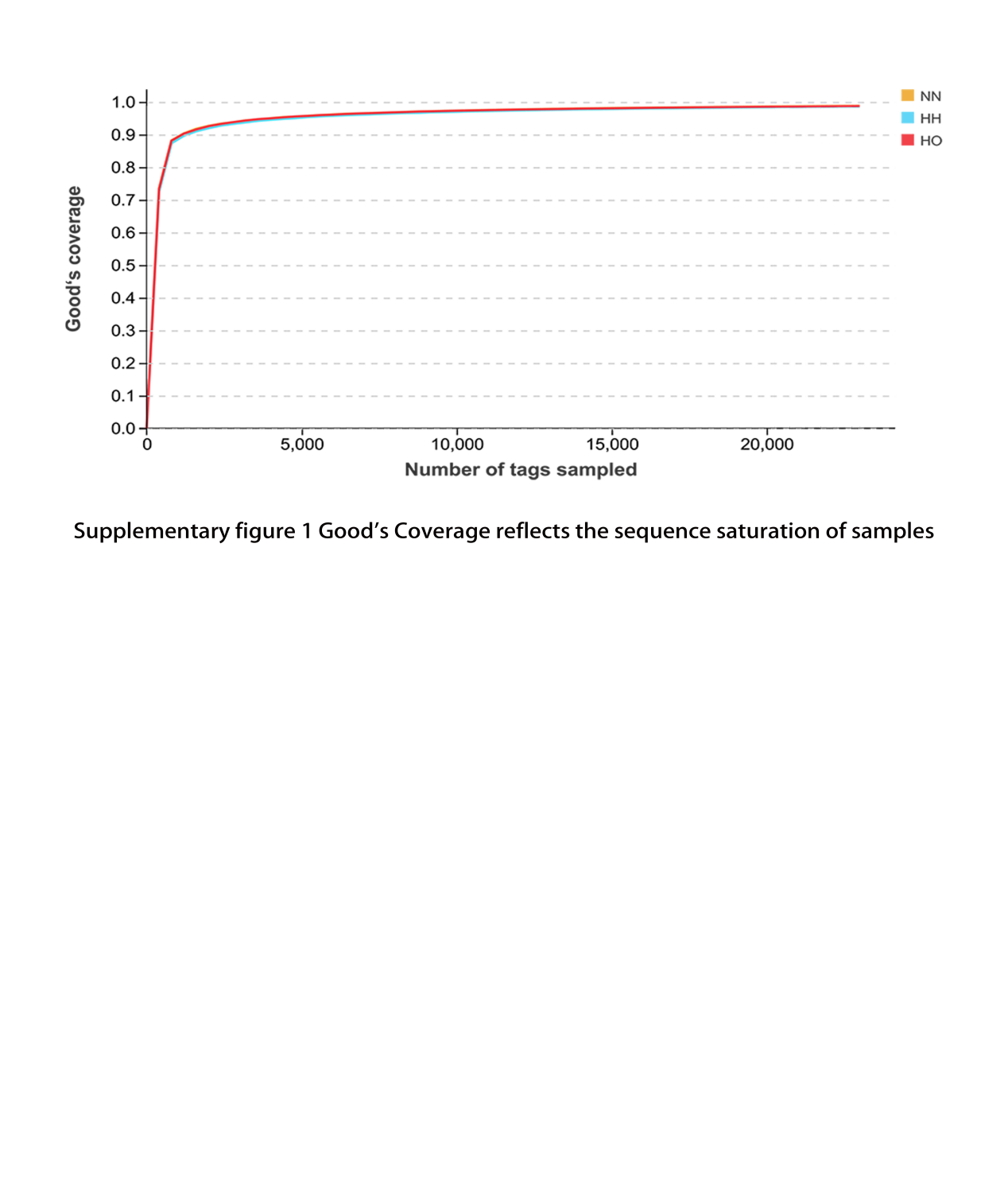


Figure 1 Good's Coverage reflects the sequence saturation of samples.

Supplement: Supplementary file 5 [file Data_Sheet_1.docx]
